# Bœnninghausen’s Aphorisms of Hippocrates

**Published:** 1887-03

**Authors:** 


### BŒNNINGHAUSEN'S APHORISMS OF HIPPOCRATES.

EXTRACTS TRANSLATED BY DR. A. McNEIL, SAN FRANCISCO.

### HYDROPHOBIA.

In our long practice we have had only two cases of fully developed hydrophobia. The first is in the *Archiv für Hom. Heilkunde*, Band X, given in detail. The patient had been already treated allopathically, both internally and externally, and thus overdosed; yet he was quickly and completely cured. The second was in 1854, when there was an unusual number of rabid dogs. In the last three years, *notwithstanding* the great heat, scarcely any have been seen. This case was a countryman in the prime of life, who had been bitten seven days before in the hand by a rabid dog. On this morning he perceived in his head early in the morning *on washing* the first beginning of the disease. He immediately came to us for aid. While we were examining him in our office, he was attacked by the second paroxysm, placing us in an unpleasant situation. It began with a painful *drawing from the neck* to the forehead, then immediately *sparks* before the eyes and entire *loss of sight, with red face* and involuntary *gnashing of the teeth.* He was immediately led to a seat reclining backward and requested to keep quiet, which he was still able to do. In five minutes the paroxysm was over, and he was immediately given a minimum dose of Bellad.$^{200}$, on the tongue, and afterward two other doses of the same, and two interpolated doses of Hyoscy.$^{200}$, and was ordered to take one every twenty-four hours. The result was perfect. On the sixth day he returned and assured us that he had not *had the slightest hint* of the disease. But as the wound on his hand revealed a *bluish* color shining through, he was given Bell. powders with Lachesis interpolated, instead of Hyos., on account of the *blue color.*

When he returned in eight days, all was healed and of natural color, and not the least inclination of ill health discoverable, and up to this time he rejoices in perfect health.

The prophylactic procedures of the two rival schools correspond perfectly to the above views and modes of treatment. While allopathy cuts, corrodes, and burns, which certainly no one can call jocund, Homœopathy gives simply and quietly her little white, sweet powders, which contain the medicines in the higher dynamizations which have the greatest similarity in their effects to the usual signs of hydrophobia. Among these, Belladonna stands at the head, and in order to make its efficacy more certain we choose only a high dynamization, by which, according to our experience, the sphere of action of a medicinal substance *is much enlarged;* but we also give as an intercurrent remedy, at one time Hyoscy. and another Stramon., because these remedies, besides their partial symptomatic similarity to that of the true hydrophobia, have the propensity of rendering the organism more susceptible to the action of Belladonna. This procedure has, in fact, *proven itself so successful*, that in a wide region nine out of ten who are now bitten by mad dogs seek our aid, and of the *hundreds* who have used our prophylactics *not one has had an outbreak* of the frightful disease, or even the slightest attack.

